# The Role of ERRFI1+808T/G Polymorphism in Diabetic Nephropathy

**DOI:** 10.22088/IJMCM.BUMS.8.2.49

**Published:** 2019-06-15

**Authors:** Saeedeh Asgarbeik, Mahsa Mohammad Amoli, Samaneh Enayati, Fatemeh Bandarian, Ensieh Nasli-Esfahani, Katayoon Forouzanfar, Farideh Razi, Seyed Abdolhamid Angaji

**Affiliations:** 1 *Department of Cellular and Molecular Biology, Faculty of Biological Sciences, Kharazmi University, Tehran, Iran.*; 2 *Metabolic Disorders Research Center, Endocrinology and Metabolism Molecular-Cellular Sciences Institute, Tehran University of Medical Sciences, Tehran, Iran.*; 3 *Endocrinology and Metabolism Research Center, Endocrinology and Metabolism Clinical Sciences Institute, Tehran University of Medical Sciences, Tehran, Iran.*; 4 *Personalized Medicine Research Center, Endocrinology and Metabolism Clinical Sciences Institute, Tehran University of Medical Sciences, Tehran, Iran.*; 5 *Diabetes Research Center, Endocrinology and Metabolism Clinical Sciences Institute, Tehran University of Medical Sciences, Tehran, Iran.*; 6 *Elderly Health Research Center, Endocrinology and Metabolism Population Sciences Institute, Tehran University of Medical Sciences, Tehran, Iran.*; 7 *Diabetes Research Center, Endocrinology and Metabolism Clinical Sciences Institute, Tehran University of Medical Sciences, Tehran, Iran.*

**Keywords:** ERRFI1, +808T/G, polymorphism, rs397349, diabetic nephropathy

## Abstract

Nephropathy is a common diabetes complication. *ERRFI1* gene which participates in various cellular pathways has been proposed as a candidate gene in diabetic nephropathy. This study aimed to investigate the role of +808T/G polymorphism (rs377349) in *ERRFI1* gene in diabetic nephropathy. In this case-control study, patients including diabetes with nephropathy (DN=104), type 2 diabetes without nephropathy (DM=100), and healthy controls (HC=106) were included. DNA was extracted from blood, and genotyping of the +808T/G polymorphism was carried out using PCR-RFLP technique. The differences for genotype and allele frequencies for +808T/G polymorphism in *ERRFI1* gene between DN vs. HC and DN+DM vs. HC were significant (P<0.05) while no significant difference between DN and DM was observed. The allele frequencies were significantly different in DN vs. HC and DN+DM vs. HC in males but not in females. G allele of +808T/G polymorphism in *ERRFI1* gene has no significant role in development and progression of diabetic nephropathy in diabetes patients while it is a risk allele for developing diabetes in Iranian population.

Diabetes is a common chronic metabolic disorder associated with different macro and microvascular complications that leads to long-term damage and various organs failure. Diabetes complications have been widely studied previously in Iran, and research gaps have been identified and roadmap has been drawn (1, 2). Diabetic nephropathy (DN) is a common and serious microvascular complication of diabetes and affects about 30-40 percent of patients with diabetes (3). Diabetes is the leading cause of chronic kidney disease and end stage renal disease (ESRD) (4). The death rate of kidney disease is 17 times higher in individuals with diabetes than non-diabetics (5). However, even with intensive glucose and blood pressure control, over 1/3 of diabetes patients develop DN (6, 7) which highlights the role of genetic factors. Individuals with affected parents have a greater susceptibility to diabetic nephropathy than unaffected ones (8). Up to now, different studies have investigated the association of various genetic factors such as engulfment and cell motility protein 1 (*ELMO1*), apolipoprotein E (*APOE*) and myosin 9 (*MYH9*) gene variants with DN (9) (10-13).

ERBB receptor feedback inhibitor 1 (*ERRFI1*) gene is located on 1p36.23 and consists of 5 exons. *ERRFI1 *or so-called receptor-associated late transducer (*RALT*), *GENE-33* or mitogen-inducible gene 6 (*MIG-6*) which is involved in various signaling pathways, has been proposed as a target gene in DN (14, 15). The *ERRFI1* gene encodes an adapter protein which regulates tyrosine kinase receptors such as epidermal growth factor receptor (EGFR), cell division control protein 42 (Cdc42)/ Rac-interaction and binding (CRIB) domain, Src homology-3(SH3)14-3-3 binding domain and MET proto oncogene (16-18). The expression of *ERRFI1* gene is rapidly increased due to stress, mechanical strain and the presence of various hormones and growth factors, which finally leads to hypertrophy (15-17, 19, 20). This gene has a responsive element to insulin, and its expression is elevated in diabetes (21, 22). The *ERRFI1* gene has high expression in the liver and kidney, and has a moderate to low expression in brain, lung, placenta, heart, and thymus tissues (23). Increasing the expression of *ERRFI1* in beta cells results in apoptosis and cell death (24). ERRFI1 seems to reduce the mass of beta cells through its antagonistic role in the EGFR signaling pathway (24, 25). *ERRFI1* gene transcription is enhanced by stress-activated protein kinases (SAPKs) which can lead to hypertrophy and progression towards nephropathy (15). There is a mutual relationship between SAPKs and *ERRFI1* gene. In nephropathy, SAPKs activate *ERRFI1* transcription and activated ERRFI1 triggers the stability and expression of *SAPKs* (15). The +808 (T/G) (rs397349) variant is located in the third intron of the downstream transcription site of *ERRFI1* gene (20). The association of *ERRFI1* gene polymorphism with fasting plasma glucose and its protective effect in DN has been reported (20). The purpose of the present study was to investigate the association between +808 (T/G) polymorphism and DN in a group of Iranian diabetic patients.

## Materials and methods

**Subjects**

Subjects comprised 100 type 2 diabetes mellitus patients without nephropathy (DM), 104 type 2 diabetes mellitus patients with diabetic nephropathy (DN), and 106 individuals as healthy control (HC) that were selected from the Diabetes and Metabolic Diseases Clinic of Dr. Shariati Hospital Tehran, Iran between 2015-2017. DN patients consisted of those with more than five years history of type 2 diabetes mellitus and increased urinary albumin excretion (>30 mg/g creatinine confirmed by repeated analysis within 3-6 months). A group of diabetic patients without albuminuria and a group of healthy people without history of diabetes and kidney disease were also included in this study. Detailed medical history and informed consents were obtained from all individuals. Demographic information such as age, duration of diabetes, body mass index (BMI), and gender was also recorded. Systolic blood pressure (SBP), diastolic blood pressure (DBP), fasting blood sugar, urea, uric acid, creatinine, and urine albumin to creatinine ratio (ACR), was measured by commercial kits (Pars Azmun, Karaj, Iran). HbA1C was measured by high-performance liquid chromatography (HPLC) method (TOSOH G8, Tokyo, Japan). Glomerular filtration rate (GFR) was estimated by Cockcroft-gault equation. Exclusion criteria were HbA1c> 9%, urinary tract infection, uncontrolled high blood pressure, heart failure, pregnancy, acute septic infection, smoking, hematuria, and heavy exercise. This study was approved by the Ethics Committee of the Endocrinology and Metabolism Research Institute (EMRI) of Tehran University of Medical Sciences.

**Molecular analysis**

Total genomic DNA was isolated by the phenol-chloroform method. The region encompassing *ERRFI1* gene +808 variant was amplified by PCR with forward 5'TGAACACA TTGCAGGCAAGTCC3' and reverse 5'GCCTTG CCATTTAAGACATGCG3' primers, and the amplification product was visualized on 2% agarose gel after electrophoresis. Amplification was performed with an initial denaturation at 95 °C for 5 min, followed by 35 cycles of denaturation at 95 °C for 30 s, annealing at 63 °C for 30 s, extension at 72 °C for 40 s, with a final extension period of 5 min at 72 °C. Restriction endonuclease digestion was carried out in a 10 μl reaction solution containing 3 μl PCR products, 1 μl 10X buffer, and 0.35 μl BsuRI (*HaeIII*) (Thermo Fisher, Waltham, Massachusetts, United States) at 37°C overnight. The +808T/G conversion introduces a new BsuRI restriction site which results in cutting of the 122 bp amplicon into 76 and 46 bp fragments. Digested fragments were separated on 3% agarose gel.

**Statistical analysis**

The unpaired t-test was used to compare mean ± SD of clinical and biochemical data between studied groups. Fisher's exact test was used for calculating hardy Weinberg equilibrium for small numbers (26). Odds ratios (OR) and 95% confidence intervals (CI) were calculated using chi-square test. P-value <0.05 was considered statistically significant. All statistical analyses were performed by SPSS software version 16.

## Results

**Clinical and biochemical characteristics**

Clinical and biochemical characteristics of studied groups are represented in Table 1. There was a significant difference in age, duration of diabetes, DBP, HbA1c, urea, creatinine, ACR, and GFR between DN and DM groups (P<0.05). When HC and DN groups were compared, there were statistical differences in age, BMI, SBP, DBP, FBS, HbA1c, urea, creatinine, ACR, GFR between two groups (P<0.05). The age, BMI, SBP, FBS, HbA1c, urea and creatinine was significantly different between DM and HC groups (P<0.05).

**Genotypes and alleles distribution**

The distribution of genotypes and alleles is shown in (Table 2). Allele and genotype frequencies conform the Hardy-Weinberg equilibrium in controls (P=0.5). The genotype distribution were significantly different between DN and HC (P=0.02) and (DN+DM) *vs.* HC (P=0.04). There was no significant difference between DN and DM groups (P=0.67). The allele frequencies were also significantly different between DN and HC groups (P=0.02) and (DN+DM) *vs.* HC (P= 0.02), in contrast to DN *vs.* DM (P=0.63).

We found a gender specific association in different groups (Table 3). There was no difference in allele frequencies between females of DN *vs.* DM (P = 0.69), DM *vs. *HC (P = 0.62) and (DN+DM) *vs.* HC (P = 0.37). In male group, there was a significant difference for allele frequencies between DN *vs. *HC group (P *= *0.02) and DN+DM *vs.* HC (P = 0.03). The allele frequencies were not significantly different between males of DN and. DM groups (P = 0.44).

**Table 1:** Clinical and biochemical test results

	**HC** **(N=106)**	**DM** **(N=100)**	**DN** **(N=104)**
	**Mean ** **±** **SD**	**Mean ** **± ** **SD**	**Mean ** **± ** **SD**
**Gender (female/male)**	67/38	54/46	39/64
**Age (years)**	50.37±10.64	57.49 ±8.24 (c)	62.49±9.20 (a, b)
**Duration of diabetes (years)**	-	10.18 ±6.13	13.29±7.85 (a)
**BMI (Kg/m** ^2^ **)**	27.15±4.25	28.48±4.20 (c)	29.66±4.91 (b)
**SBP (mmHg)**	117.01±15.82	122.85±12.41(c)	123.76±13.77 (b)
**DBP (mmHg)**	79.84±9.11	78.95±6.36	75.50±9.75 (a, b)
**FBS (mg/dl)**	90.12±10.13	140.22±34.69 (c)	133.86±45.95 (b)
**HbA1C (%)**	5.50±0.47	7.10±0.74 (c)	7.39±0.74 (a, b)
**Urea (mg/dl)**	29.83±7.78	36.76±13.07 (c)	42.60±16.97 (a, b)
**Creatinine (mg/dl)**	0.99±0.16	1.11±0.25 (c)	1.27±0.48 (a, b)
**Uric Acid (mg/dl)**	4.93±2.97	5.25±1.37	5.43±1.77
**ACR (mg/grcr)**	23.09±8.31	24.42±5.96	246.67±666.55 (a, b)
**GFR (l/min/1.73 m** ^2^ **)**	77.41±28.84	76.73±20.0	64.36±29.76 (a, b)


**Table 2:** Frequencies of +808T/G genotypes and alleles

	**DN** **(n=104)** **n(%)**	**DM** **(n=100)** **n(%)**	**DN+DM** **(n=204)** **n(%)**	**HC** **(n=106)** **n(%)**
**Genotype**				
**TT**	89 (85.58%)	90 (90%)	179 (87.75%	100 (94.33%)
**GT**	14 (13.46%)	7 (7%)	21 (10.29%)	6 (5.67%)
**GG**	1 (0.96%)	3 (3%)	4 (1.96%)	0
$x^{2}$		0.17	4.04	4.78
**P** ***-*** ** value**		0.67	0.04*	0.02*
**Allele**				
**T**	192 (92.31%)	187 (93.5%)	379 (92.89%)	206 (97.17%)
**G**	16 (7.69%)	13 (6.5%)	29 (7.11%)	6 (2.83%)
**P** ***-*** ** value**		0.63	0.02*	0.02*
**OR (95% CI)**		0.83 (0.39-1.78)	0.38 (0.15-0.93)	0.35 (0.13-0.91)

**Table 3.** Allele frequencies according to gender

		**DN**	**DM**	**DN+DM**	**HC**
**Female**	**G**	4 (5.13%)	7 (6.48%)	11 (5.91%)	5 (3.73%)
	**T**	74 (94.87%)	101 (93.52%)	175 (94.09%)	129 (96.27%)
**P** ***-*** **value**			0.69	0.37	0.62
**OR (95% CI)**			1.28 (0.36-4.54)	0.61 (0.20-1.81)	0.71 (0.18-2.75)
**Male**	**G**	12 (9.38%)	6 (6.52%)	18 (8.18%)	1 (1.32%)
	**T**	116 (90.62%)	86 (93.48%)	202 (91.82%)	75 (98.68%)
**P** ***-*** **value**			0.44	0.03*	0.02*
**OR (95% CI)**			0.67 (0.24-1.86)	0.15 (0.02-1.14)	0.12 (0.01-1.01)

**Table 4:** Clinical and biochemical tests in diabetes groups with or without nephropathy

	**GG+TG** **(N=25)**	**TT ** **(N=179)**	**P-** ***v*** **alue**
	Mean ± SD	Mean ± SD	
**Age (years)**	61.80±11.22	59.76±8.73	0.29
**Duration of diabetes (years)**	13.76± 9.04	11.46± 6.89	0.13
**BMI (Kg/m** ^2^ **)**	29.86 ±4.18	28.93± 4.64	0.35
**SBP (mmHg)**	119.37 ±18.07	123.84 ±12.22	0.25
**DBP (mmHg)**	74.79± 10.37	77.55± 8.07	0.22
**FBS (mg/dl)**	141.64 ±40.03	136.32 ±41.03	0.54
**HbA1c (%)**	7.19±0.62	7.25 ±.77	0.70
**Urea (mg/dl)**	44.0± 19.81	39.10 ±14.66	0.25
**Creatinine (mg/dl)**	1.19 ±.33	1.19 ±.40	0.97
**Uric acid (mg/dl)**	5.52± 1.81	5.29± 1.51	0.53
**ACR (mg/grcr)**	112.91±170.82	141.19± 516.93	0.78
**GFR (l/min/1.73 m** ^2^ **)**	70.0 ±25.45	70.55±26.25	0.92


Then DN and DM groups were pooled together, and clinical and biochemical parameters between individuals with GG+GT genotypes (allele G) and TT genotype (allele T) were compared (Table 4). The age, duration of diabetes, BMI, FBS, urea and uric acid were higher, and SBP, DBP, A1c and ACR were lower in GG+GT group in comparison with TT group, although it was not statistically significant (P> 0.05). There was no significant difference in any biochemical parameters between the two groups with GG+GT and TT genotypes.

## Discussion

The results of the current study showed a significant association between +808T/G variant and diabetes but not DN. The study showed a significant difference in genotype and allele frequencies between DN *vs*. HC and DN+DM *vs.* HC group (P <0.05).

The study of Li et al. showed that the attenuated expression of the *ERRFI1* gene lead to sustained activation of the EGFR pathway, and migration and proliferation of cells in  non-small cell lung cancer (NSCLC) (27). In another study, the role of MIG-6*ERRFI1* gene in neointima formation in cardiovascular disease was investigated, and it was shown that the artery Mig-6 knocked down mouse had a 5.3 fold increase in neointima formation in comparison with the control group. Their results showed the role of MIG-6 in the progression of atherosclerosis (28). Lee et al. investigated the association of *ERRFI1* +808T/G polymorphism with diabetic nephropathy for the first time in the Korean population (20). Diabetic patients with T allele had 1.81 times more chance of developing diabetic nephropathy than those with G allele (20). No difference was found between T and G alleles in men with diabetic mellitus, while women carrying T allele showed a 2.12 fold increased chance of developing diabetic nephropathy in comparison with women carrying G allele (20). The results of their study demonstrated the protective role of G allele toward nephropathy development in type 2 diabetic patients (20). We investigated the association of rs377349 with diabetic nephropathy for the first time in Iranian population. In this study, the frequency of G allele was (7.69%), (6.5%), (2.83%), and (7.1%) in DN, DM, HC, and DM+DN groups, respectively. Subjects with T allele had lower chance of developing diabetes than those carrying G allele, and T allele was protective against diabetes. In this study although males who carried G allele in diabetes group were more prone to develop diabetic nephropathy, this difference was not significant. There was no difference in allele frequencies in female groups. In a study on Korean population, it was claimed that individuals with G allele had a higher level of fasting plasma glucose, and lower level of ACR in comparison with those carrying T allele (20). In the present study, although in individuals carrying G allele (GG+GT) the amount of FBS was higher and ACR was lower than those carrying T allele (TT), the difference did not reach significance (P > 0.05). As the frequencies of G allele in DN, DM and DN+DM groups were higher than the HC group, in disagreement to the previous studies, we found evidence about the protective role of T allele against type 2 diabetes. In other words, the results of this study indicate that G allele may not play a role in the development and progression of diabetic nephropathy in type 2 diabetic patients. On the other hand, G allele may be considered as a risk allele instead of protective in diabetic nephropathy. The association of G allele with diabetic nephropathy did not reach significance level, although it may reach significance in other studies with greater sample size. Since the association of this polymorphism with nephropathy has been recently studied in the Korean population, there are not enough data for comparison with other populations. This is the first time that the association of this polymorphism with nephropathy has been studied in the Iranian population.

In conclusion, there was no significant association between *ERRFI1* +808T/G polymor-phism and diabetic nephropathy in Iranian population while T allele of this polymorphism has protective role against diabetes in the studied population. It seems that *ERRFI1* polymorphism has diverse function in various populations which might be due to various clinical manifestations or pathogenesis in different ethnic groups. 

It will be interesting to investigate the relationship between this polymorphism with diabetes nephropathy in other populations. 

## Conflict of interest

The authors declare no conflict of interest.
